# Work stress associated cool down reactions among nurses and hospital physicians and their relation to burnout symptoms

**DOI:** 10.1186/s12913-017-2445-3

**Published:** 2017-08-10

**Authors:** Arndt Büssing, Zarah Falkenberg, Carina Schoppe, Daniela Rodrigues Recchia, Désirée Poier

**Affiliations:** Quality of Life, Spirituality and Coping, Institute of Integrative Medicine, Witten/Herdecke University, Gerhard-Kienle-Weg 4, D-58313 Herdecke, Germany

**Keywords:** Nurses, Physicians, Patient interaction, Cool down reactions, Burnout, Stress perception, Survey

## Abstract

**Background:**

Hospital staff experience high level of work stress and they have to find strategies to adapt and react to it. When they perceive emotional exhaustion and job dissatisfaction in response to constant work stress, one reaction might be emotional withdrawal. This emotional distancing can be seen as an adaptive strategy to keep ‘functionality’ in the job. Both, perception of emotional exhaustion and emotional distancing as a strategy, can be operationalized as ‘Cool Down’. We assume that work stress associated variables are positively associated with Cool Down reactions, while internal and external resources are negatively associated and might function as a buffer against emotional distancing. Moreover, we assume that the perception of stress and work burden might be different between nurses and physicians and women and men, but not their cool down reactions as a strategy.

**Methods:**

Anonymous cross-sectional survey with standardized instruments among 1384 health care professionals (66% nurses, 34% hospital physicians). Analyses of variance, correlation and also stepwise regression analyses were performed to analyze the influence of demands and resources on Cool Down reactions.

**Results:**

As measured with the Cool Down Index (CDI), frequency and strength of Cool Down reactions did not significantly differ between women and men, while women and men differ significantly for their burnout symptoms, stress perception and perceived work burden. With respect to profession, Cool Down and stress perception were not significantly different, but burnout and work burden. For nurses, “Emotional Exhaustion” was the best CDI predictor (51% explained variance), while in physicians it was “Depersonalization” (44% explained variance). Among putative resources which might buffer against Cool Down reactions, only team satisfaction and situational awareness had some influence, but not self-efficacy expectation.

**Conclusion:**

The perceptions of emotional exhaustion and distancing of nurses and physicians (and women and men) seems to be different, but not their adaptive Cool Down reactions. Data would support the notion that a structural approach of support would require first to control and eliminate work stressors, and second a multifaceted approach to strengthen and support hospital staff’s resources and resilience.

**Electronic supplementary material:**

The online version of this article (doi:10.1186/s12913-017-2445-3) contains supplementary material, which is available to authorized users.

## Background

Health care professionals experience an increasing work burden, resulting often in states of emotional exhaustion / burnout symptoms [1, 2]. The underlying causes are manifold; among them are requirements of extensive documentation and thus reduced time for patient encounter, and pressure to reduce health care costs and hospital stays [3–5]. Health care professionals have to deal with high staff turnover rates, heavy workloads, staffing shortages, shift working and working at night, which contribute to higher stress levels [1, 6]. A higher stress potential in the hospital personnel may derive from high demanding moral and emotional activities and by a high level of responsibility [2, 7, 8]. An increased patient acuity and a high number of multimorbid patients also contribute to an experience of increasing work burden by health care professionals, who have to deal “with the most emotionally distressing of situations-illness, dying, suffering in every form” [9].

Among the predominating models referring to the development of high stress potential and mental strain [10–14], the *Job Demand-Resource model* is suited to explain the core concepts of our intention. Adverse working conditions and perceived work stress (as demands) are mostly associated with poor psychological wellbeing, emotional exhaustion, reduced life satisfaction, dissatisfaction with the job and lower quality of work, etc. – or even with the decision to quit the job [15–18]. Such work-associated stress may result in the classical triad of burnout symptoms as described by Maslach et al. [19], i.e., emotional exhaustion, depersonalization, and reduced personal accomplishment, and finally disability to work. McManus et al., [20] laconically stated that “burnout and stress are common, linked problems in health-care workers”. In their study among physicians from the United Kingdom they observed reciprocal causation between emotional exhaustion and stress [20].

It is clear that all health care professionals have to cope with work stress and have to find strategies to adapt and to stay ‘functional’ while doing their job. They “learn to distance themselves from recipients in order to help them better” [21]. If this professional skill is not adequately developed, it might lead to ‘depersonalization’ which is described by Schaufeli and Buunk [21] as one possible strategy of health care professionals to cope with emotional exhaustion. It describes the development of an “impersonal, negative, callous and cynical attitude” towards the patients [21]. Another strategy might be the development of emotional distance towards the patients [21]. Research has underlined that the everyday adverse and stressful working conditions are “significant barriers to compassionate care” [22] with a negative impact on the development of empathy in practicing physicians [6].

For our research we focus on emotional distancing or withdrawal related to health professionals’ perceived emotional exhaustion as the result of their work stress, which can be labeled as ‘Cool Down’ with respect to the carer-patient relationship.

### Cool down reactions – Conceptual considerations

**Fig. 1:**
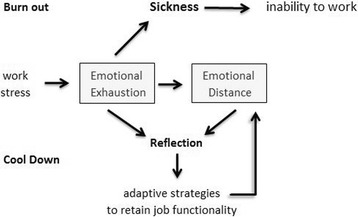
Schematic representation of cool down reactions and burnout as a process

Conceptually, ‘Cool Down’ reactions imply two steps: 1) a person’s own *perception* of an emotional exhaustion in response to work stress, and 2) active attempts to find *adaptive strategies* in terms of emotional distancing to retain ‘functionality’ in the job [23, 24] instead of dysfunctional exhaustion as assumed for burnout. Despite conceptual similarities between Cool Down and burnout in the first phases (Fig. 1), the individual reactions may result in different *end-points*, i.e., either times of sickness and finally inability to work (burnout), or finding active strategies to keep ‘functionality’ in the job (cool down) which requires a *reflection process* and a decision to emotionally withdraw from the patients as a coping strategy. Cool down is clearly seen as a strategy to continue performing the work duties, but with a negative impact on emotional care for the patients [23, 24]. Affected work behavior (“Personal accomplishment”) is not part of the Cool Down concept, but an integral part of Maslach’s burnout concept [9, 25].

During their education nurses, medical students and physicians are often advised by experienced superiors - or they learn it on role models - to distance themselves from the suffering of their patients to protect the own emotional stability and functionality [24]. They “learn to distance themselves from recipients in order to help them better”, as others have argued, too [21]. Insofar, this strategy is ‘useful’ only for the health professionals. However, the consequence of this emotional distancing will be care as a ‘duty’, and probably lower quality of care, too. Besides a good technical performance, which is the prerequisite of professional care, the interpersonal performance is of outstanding relevance for the quality of care, i.e., the empathic and helping relationship, being focused on the patients’ needs and problems, the unseparated and attending time spend with them, respectful communication, etc. [26]. Thus, Cool Down reactions will address specifically this point of *impaired personal relationship* which is a consequence of a person’s strategic decision to actively cope with perceived emotional exhaustion, resulting in emotional distancing. While these individual (and relation-oriented) reactions are crucial for the Cool Down concept, burnout is mostly due to environmental factors which belong to the work setting and the inability to cope with those stressors of daily life [25]. These would result in depressive symptoms, substance abuse and potentially loss of job (Fig. 1). Apart from cynicism, adaptive strategies are not addressed in the Maslach’s Burnout Inventory (MBI).

### Cool down and related research hypotheses

**Fig. 2:**
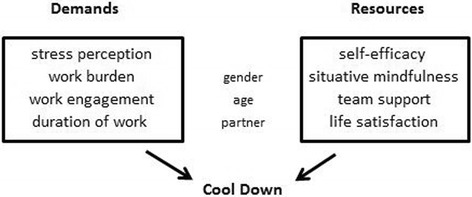
Influence of job related demands (stressors) and available resources (buffers) on Cool Down reactions

With respect to a modified *Job Demand – Resource model*, Cool Down reactions – as measured with the Cool Down Index [24] – may have their causes in specific demands (i.e., *aggravating factors* such as work burden, stress perception, and duration of work) and are probably buffered by individual resources (i.e., *protective factors* such as self-efficacy expectation, situational awareness / mindfulness, team support and partnership) (Fig. 2).

Among the protective variables we see health professionals’ self-efficacy expectation which refers to a person’s ability to reach specific goals, aims or tasks, or more precisely to the beliefs about the own capabilities and conviction that one can successfully deal with / manage their responses. It is expected that a person copes better with adversive experiences or challenging tasks (such as work load or work stress) when their self-efficacy expectation is high [27]. Therefore, one may suppose that Cool Down reactions should be lower when health professionals have higher expectations of personal efficacy.

A further resource might be a persons’ situational awareness (‘mindfulness’), because it is regarded as helpful to build teamwork and to promote compassion. In healthy (non-clinical) individuals, a recent meta-analysis has confirmed “large effects on stress, moderate effects on anxiety, depression, distress, and quality of life, and small effects on burnout” for mindfulness programs [28]. In nurses, mindfulness-based stress reduction applied as an intervention program improved their coping with work stress (i.e., lower stress, burnout and anxiety, higher empathy, focus and mood) [29]. In line with this, we assume that health professionals’ (‘natural’) situational awareness (which is different from the ‘trained’ mindfulness of respective programs) can buffer their stress related Cool Down reactions.

Health care professionals’ work engagement and also life satisfaction are seen as ambivalent variables. They can be either positive variables with respect to Cool Down reactions when work is perceived as a fulfilling activity - characterized by vigor, dedication and absorption [30] - and when life in general is perceived as satisfying; or a negative variable when work engagement is low because of high work burden, stress and duration of work or when life in general is seen as less satisfying. It has been shown that work engagement is negatively associated with burnout [31], and positively related to work and life satisfaction and self-rated health [30]. In this context we see work engagement and life satisfaction as positive variables. Satisfaction with the hospital team (‘team support’) is regarded as a Cool Down buffering variable, because van der Doef and Maes [14] reported that social support may buffer some of the negative influence of given stressors. In the same vein we see partner support as a buffering variable.

### Aim of the study and underlying hypotheses

One may assume that the hospital staff is aware of their work stress, and that they are able to reflect on their emotional exhaustion; some of them may decide to distance themselves from their patients. One may also assume that most are not satisfied with these emotional withdrawal reactions because they do know that this will affect the caring relation with the patient and their satisfaction with own work, too. Therefore, we intended to measure these perceptions, particularly to identify which persons among the hospital staff experience Cool Down, and further to analyze Cool Down aggravating and buffering variables as depicted in Fig. 2. We assume that 1) all hospital staff (differentiated with respect to profession and gender) experience Cool Down to a similar degree, but 2) that the perception of stress and work burden (incl. burnout symptoms) might be different between nurses and physicians and women and men, but not their Cool down reactions which are seen as an adaptive strategy. Further, we assume that 3) work stress associated variables (i.e., perceived stress and work burden, duration of work per week, and burnout symptoms) are positively associated with Cool down reactions, while internal and external resources (i.e., self-efficacy expectation, situational awareness, partner support, team satisfaction) are negatively associated and might function as a buffer. Age was tested as a further variable because one may assume that persons with higher age may have found strategies to cope with work stress, while younger persons might thus be more vulnerable to work stress.

## Methods

### Participants

This anonymous cross-sectional study (survey) was performed with two sample populations, i.e., nurses and hospital physicians (Table 1), and approved by the IRB of the Witten/Herdecke University (#59/2013 and #25/2014). We provided an information sheet which described the background of the study, guaranteed anonymity and assured confidentiality. All had the option to respond anonymously via an internet based questionnaire or conventional paper-pencil questionnaire. Printed questionnaires were stored at the Witten/Herdecke University. None of the hospital managers has access to these questionnaires. All data were pooled and analyzed in more general categories (women versus men; nurses versus physicians), and were not analyzed for the recruiting sources (i.e., hospitals).

**Table 1:** Characteristics of the sample (*N* = 1384)

Gender	n	% or mean values
Women	945	69.5
Men	415	30.5
Age, years (mean, SD)		40.7 ± 11.3
Family status	n	%
Living with partner – married	666	49.0
Living with partner – not married	336	24.7
Single	265	19.5
Divorced	81	6.0
Widowed	10	0.7
Profession, %	n	%
Nurses	916	66.2
Physicians	467	33.8
Employment, years, (mean, SD)		16.3 ± 11.7
Duration of work per week, hours (mean, SD)		39.0 ± 12.9
Perceived work burden, 0–100 (mean, SD)		60.7 ± 21.3

Not in all cases enrolled persons provided complete information, i.e., for gender (*n* = 24), age (*n* = 155), family status (*n* = 26), employment (*n* = 32), duration of work (*n* = 59), work burden (*n* = 37)

Hospital physicians were informed about this study with information sheets administered to regional hospitals, or via website announcements of the Medical Association North-Rhine (Ärztekammer Nordrhein) and the medical union “Marburger Bund”. Among the physicians, 48% filled out the online questionnaire. Nurses of 24 predominantly regional hospitals and geriatric care units were informed with similar information sheets (85% from various regional hospitals and 15% from geriatric care units). Only a few completed the online questionnaire, while most completed the paper-pencil version. Inclusion criteria were profession as a nurse or hospital physician, being active at work, and willing to participate. We had no access to staff away sick.

All respondents were free to decide participating; there was neither an obligatory order by hospital managers to participate nor a control of completeness in requested participants. The sample should thus be regarded as a ‘convenience’ sample. To clarify whether the sample of hospital physicians may be representative for a communal hospital setting or not, we compared the data of physicians from a prevalence sample (regional hospital with a response rate of 47.5%) with those of the whole sample. Both groups did not significantly differ with respect to gender, age, family status, employment status, years of employment, duration of work per week, and perceived work burden (data not shown). Thus, with the exception of the specialization pattern we can regard the whole sample as representative for hospital physicians working in local hospitals. Because most nurses filled the paper questionnaire, we were able to calculate their response rates which ranged from 11 to 57% in the different hospitals (in average 28.5%). Nurses working for temporary employment agencies showed a response rate of 46.9%. All in all we enrolled 1384 persons (66% nurses, 34% physicians).

To describe the enrolled health professionals, we analyzed their profession, gender, age, family / partner status, time of employment, and duration of work per week.

### Measure

To measures the Cool Down and burnout symptoms and to relate these with health professional’ demands and resources (Fig. 2), the following standardized questionnaires were administered in their German language versions:

#### Cool down index

The 9-item Cool down Index (CDI; Table 2) addresses the perception of an emotional exhaustion when caring for patients, subsequent reactions of emotional withdrawal, and the reaction of ‘working to rule’ as a strategy [24]. All items were introduced by the sentence “In dealing with the people I look after (therapeutically), I notice that…”. Respondents had to judge how often they perceive the respective feelings (1 - a few times a year or less; 2 - once a month or less; 3 - a few times a month; 4 - once a week; 5 - a few times a week; 6 - every day), and with scores ranging from 1 (weak) to 6 (very strong) how strong these feeling are (Additional file 1). The sum of both scores indicates the significance of the respective feeling on a single item level (scores may range from 2 to 12), while the sum of all nine items constitutes the CDI score (scores may range from 18 to 108). In a sample of Austrian nurses the CDI had a good internal reliability (alpha = .86) [23]. Similarly, in a sample of German health care professionals (physicians, nurses and therapist), the CDI’s alpha was .84 [24].

**Table 2:** Mean values, reliability and factor analysis of CDI items (physicians and nurses)

CDI single items	Mean ± SD	Corrected Item - Scale Correlation	α if Item deleted (α = .87)	Loading Factor 1 (α = .83)	Loading Factor 2 (α = .80)
Factor 1: Perception of emotional distance (eigenvalue: 4.3; 34% explained variance)					
CDI 8 – some of them simply annoy me	5.8 ± 2.9	0.60	0.85	0.79	
CDI 4 – I often no longer have the patience to listen to them	5.5 ± 2.8	0.62	0.85	0.75	
CDI 10 – I increasingly ‘work to rule’	5.2 ± 3.1	0.61	0.85	0.70	
CDI 9 – I myself increasingly go short	6.1 ± 3.3	0.67	0.84	0.68	0.36
CDI 7 – I increasingly think how nice it would be to pack it all in	5.0 ± 3.2	0.62	0.85	0.65	0.33
CDI 5 – I largely don’t care what they think of me	4.0 ± 2.5	0.44	0.86	0.54	
Factor 2: Emotional withdrawal as strategy (eigenvalue: 1.0; 26% explained variance)					
CDI 1 – I simply must stop letting everything get to me to such an extent	6.0 ± 3.0	0.57	0.85		0.88
CDI 2 – I have to withdraw with increasing frequency to protect myself	5.6 ± 3.0	0.64	0.85		0.85
CDI 3 – their personal problems and worries often simply become too much for me	4.8 ± 2.7	0.60	0.85	0.38	0.65

Extraction of the main components (Eigenvalue >1); varimax rotation with Kaiser’s normalization. Kaiser-Mayer-Olkin value = 0.88. Factors explain 60% of variance. Factor loadings < .3 were not depicted

#### Maslach burnout inventory

To measure health professionals’ burnout symptoms, we used Maslach’s Burnout Inventory (MBI) with its three subscales “Emotional Exhaustion”, “Depersonalization” and (lack of) “Personal Accomplishment” [19]. “Emotional Exhaustion” refers to the rather depressive feelings of being overextended and exhausted by the work duties; “Depersonalization” refers to unfeeling and impersonal responses towards others; “Personal Accomplishment” refers to feelings of competence and successful achievement in the work with others [19]. The 22 items measure how often the respective feelings and perceptions may appear, and were scored from 1 (a few times a year or less) to 6 (every day). To calculate the MBI sum score, the items of the “Personal Accomplishment” sub-scale were recoded (and would thus indicate a lack of personal accomplishment), while they were used non-recoded for the respective subscale (and would thus indicate a positive personal accomplishment). In this sample, the alpha coefficients of the subscales “Emotional Exhaustion”, “Depersonalization” and “Personal Accomplishment” were .88, .74, and .79, respectively.

#### Stress perception

To assess health professionals’ stress level, we used Cohen’s Perceived Stress Scale (PSS) [32]. This instrument measures a person’s self-perceived stress level in specific situations during the last month. Four items use a reverse scoring. All items refer to emotions and thoughts, and how often one may have felt or thought a certain way. The scores range from 1 (never) to 4 (very often) and were summed. The higher the sum scores are, the higher the perceived stress is. Internal reliability of the original PSS was moderate (alpha = .78) [32]. In this sample, Cronbach’s alpha coefficient was good (alpha = .84).

#### Work burden

Self-Perceived Work Burden which is assumed to be positively related to the CDI and MBI scores, was measured with a numeric analogue scale (NAS) ranging from 0 (not at all) to 100 (extreme) [24].

#### Work engagement

Work engagement was measured with the Utrecht Work Engagement Scale (UWES) which addresses “a positive, fulfilling, work-related state of mind that is characterized by vigor, dedication, and absorption” [30]. For this study, we used the 9-item shortened version (UWES-9; alpha ranging between .85 and .92) which has similar psychometric properties as the long version. Specific items are, “At my job, I feel strong and vigorous”, “I am immersed in my work”, etc. The items are scored on a 7-point Likert scale, ranging from ‘never’ to ‘always / every day’. In this study, we did not use the subscale scores, but the combined means of all items. UWES-9’s alpha coefficient was .94 in this sample.

#### Life satisfaction

Life satisfaction was measured using the Brief Multidimensional Life Satisfaction Scale (BMLSS; alpha = .87) [33]. The items address intrinsic (myself, life in general), social (friendships, family life), external (work situation, where I live), prospective dimensions (financial situation, future prospects) of life satisfaction, and also satisfaction with the abilities to manage daily life concerns and satisfaction with the health situation. Each of these 10 items was introduced by the sentence ‘I would describe my level of satisfaction as …’, and scored on a 7-point scale ranging from dissatisfaction to satisfaction. The mean scores were referred to a 100% level (‘delighted’). In this sample, Cronbach’s alpha of the 10 item scale was .87.

The scale was complemented by five additional items, which were used as an independent measure addressing satisfaction with the team, i.e., support by colleagues and superiors, appreciation by colleagues and superiors, and with the team spirit. These five items collapse into a single “Satisfaction with Team” scale that showed a good internal consistency in this sample (Cronbach’s alpha = .89).

#### Self-efficacy expectation

Because one may suggest that particularly individuals with high self-efficacy expectations are more able to manage stress and work load, we assessed self-efficacy exspectation of nurses and physicians with the General Self-Efficacy Scale (SES) by Schwarzer & Jerusalem [34]. The 10 items can be answered on a 4-point Likert scale ranging from disagreement to agreement. One assigns values 1 to 4 which are then transferred into scores ranging from 10 to 40. The Self-Efficacy Scale scores are sum scores; the higher the scores are, the higher (optimistic) self-efficacy was expressed. The Self-Efficacy Scale was reported to have a good to very good internal consistency, i.e., Cronbach’s alpha in German samples range from .80 to .90 [35]. In this sample, the alpha coefficient of the scale was .88.

#### Conscious presence and self control

To measure situational awareness and presence, we referred to a modified ‘mindfulness’ instrument, the Conscious Presence and Self Control (CPSC) scale [36], which refers to the 14-item Freiburg Mindfulness Inventory (FMI) by Walach et al. [37]. Because several of the primary item phrasings were less appropriate for individuals who are not familiar with mindfulness training and resulting unfamiliarity of the underlying concepts, the instrument was specified and adjusted to measure a person’s conscious presence and perception of a given situation and their self-control in difficult situations [36]. The 10-item instrument had a good internal consistency (Cronbach’s alpha = .86), and showed sound associations with measures of health affections and life satisfaction [36]. Response options were ‘rarely’ (0), ‘occasionally’ (1), ‘fairly often’ (2), and ‘almost always’ (3). Data are given as mean scores. In this sample, the alpha coefficient of the scale was .82.

### Statistical analysis

Descriptive statistics, reliability (Cronbach’s coefficient α) and factor analyses (principal component analysis using Varimax rotation with Kaiser’s normalization) of Cool Down items, as well as analyses of variance (ANOVA) of Cool Down and burnout scores within the sample, first order correlations between Cool Down and demand and resource variables, and stepwise regression analyses to identify significant Cool Down predictors were computed with SPSS 22.0. To handle missing data, we chose the MissForest method (software R) - nonparametric missing value estimation for mixed data [38], because the dataset contains both continuous and categorical variables (mixed).

Given the exploratory character of this study, significance level was set at *p* < .05. With respect to classifying the strength of the observed correlations, we regarded *r* > .5 as a strong correlation, an r between .3 and .5 as a moderate correlation, an r between .2 and .3 as a weak correlation, and *r* < .2 as a negligible correlation.

## Results

### Description of the sample

Among the 1384 enrolled persons (66% nurses, 34% physicians), 69.5% were women and 30.5% men (Table 1). Among the nurses, 80.9% were female and 19.1% male, while among the physicians, 47.5% were female and 52.5% male (*p* < .0001; Chi^2^). The mean age of nurses and physicians was similar (40.6 ± 11.6 and 40.9 ± 10.7, not significant). Duration of work per week was in average 39 ± 13 h. Mean score of self-perceived work burden was 61 ± 21, indicating moderate to stronger work burden. All further data are given in Table 1.

### Reliability and factor analysis of cool down index items

First we intended to confirm the validity of the 9-items of the CDI (since its development, item #6 is not used at all) which in fact had a good internal consistency also in this sample (Cronbach’s alpha = 0.87) (Table 2). Exploratory factor analysis pointed to two sub-constructs which accounted for 60% of variance (Table 2), i.e., the 6-item factor “Perceived Emotional Distance” (PED; alpha = .83), and the 3-item factor “Emotional Withdrawal as a Strategy” (EWS; alpha = .80). Although the instrument is intended to be used as an index, data with the respective factors (which may add specific information for the interpretations) were addressed in the further analyses.

### Factor analysis of cool down and burnout items

Because the CDI was strongly associated with the MBI (*r* = .54), we performed an explorative factor analysis to clarify whether both instruments cover the same topics or differential aspects. Therefore, we used both instruments’ items measuring the *frequency* of perceived feelings (MBI’s “Personal Accomplishment” items were not used). Exploratory factor analysis indicated 4 main factors (58% of explained variance): Factor 1 representing MBI’s “Emotional Exhaustion” subscale and two CDI items (CDI9 - I myself increasingly go short; CDI7 - I increasingly think how nice it would be to pack it all in). Factor 2 was MBI’s “Depersonalization” subscale with two of MBI’s “Emotional Exhaustion” items (MBI6 and MBI16). Factor 3 was represented by CDI’s “Perceived Emotional Distance” items with one of MBI’s “Depersonalization” item (MBI11 – I worry that this job is hardening me emotionally). Factor 4 comprises the CDI’s “Emotional Withdrawal as a Strategy” items only. Thus, the respective CDI and MBI subscales do not strongly intermix and they were differentiated as more or less independent factors.

### Correlations between cool down and demands and resources

To answer whether or not Cool down and burnout symptoms are related to health professionals’ demand and resource variables (Fig. 2), correlation analyses were performed (Table 3).

**Table 3:** Correlation between cool down sub-scales and external measures

	CDI sum	CDI - PED	CDI - EWS	MBI sum
Burnout (MBI) sum score	*.54* ^**^	*.57* ^**^	.35^**^	
MBI: Emotional Exhaustion	*.62* ^**^	*.63* ^**^	.43^**^	*.86* ^**^
MBI: Depersonalization	.49^**^	*.51* ^**^	.32^**^	*.75* ^**^
MBI: Personal Accomplishment	.19^**^	.22^**^	.10^**^	.44^**^
Stress Perception (PSS)	*.51* ^**^	*.51* ^**^	.37^**^	*.51* ^**^
Perceived Work Burden (VAS)	.45^**^	.45^**^	.33^**^	.46^**^
General Life Satisfaction (BMLSS)	−.42^**^	−.42^**^	−.32^**^	−.40^**^
Satisfaction Team Support (BMLSS-TS)	−.40^**^	−.40^**^	−.30^**^	−.37^**^
Work Engagement (UWES)	−.38^**^	−.41^**^	−.23^**^	−.45^**^
Self-Efficacy Expectation (SWE)	−.31^**^	−.30^**^	−.25^**^	−.38^**^
Conscious Presence (CPSA)	−.36^**^	−.35^**^	−.27^**^	−.40^**^
Age (years)	−.05	−.11^**^	.08^**^	−.09^**^
Duration of employment (years)	−.05	−.11^**^	.06	−.18^**^
Duration of work per week (hours)	.07	.08^**^	.03	.33^**^

*Abbreviations*: *CDI* Cool down Index, *CDI-PED* Perception of Emotional Distance, *CDI-EWS* Emotional Withdrawal as a Strategy, *MBI* Burnout

**p < .01 (Spearman-Rho); strong correlations (*r* > 0.5) were highlighted (italics)

The CDI and its sub-construct “Perceived Emotional Distance” correlated moderately to strongly with MBI’s “Emotional Exhaustion” and “Depersonalization” component on the one hand, and with stress perception and work burden on the other hand. The CDI sub-construct “Emotional Withdrawal as a Strategy” correlated much weaker and moderately with these stress-associated variables (Table 3). In contrast, MBI’s “Personal Accomplishment” correlated only marginally or weakly with the CDI and its subscales. Moreover, the CDI correlated moderately negative with health professionals’ resource variables (i.e., general life satisfaction and satisfaction with team support, work engagement, self-efficacy expectation and Conscious presence and Self-Control).

There were no significant associations between CDI and age, duration of employment or duration of work per week, while particularly duration of work per week was moderately associated with burnout symptoms (Table 3).

In the sample of nurses, we also measured their satisfaction with the own ‘quality of care’ provided. This variable was moderately negative related to CDI’s “Perceived Emotional Distance” (*r* = −.37) and only marginally with “Emotional Withdrawal as a Strategy” (*r* = −.18), with burnout symptoms (*r* = −.42) and stress perception (*r* = −.36), and moderately positive with work engagement (*r* = .42) and best with nurses’ satisfaction with the team support (*r* = .49).

### CDI, burnout and stress perception in health professionals

Next, we intended to describe the prevalence of CDI scores in the sample. The scores of the perceived *frequency* of Cool Down reactions showed a left skewness (mean: 21 ± 9; 25% percentile at 14, 75% percentile at 27; maximal score 54), while the perceived *intensity* was rather normally distributed (mean 27 ± 10; 25% percentile at 18, 75% percentile at 35; maximal score 54). Thus, the CDI (which combines *frequency* and *intensity*) showed a left skewed bulk of scores (mean: 48 ± 18; 25% percentile at 33, 75% percentile at 61; maximal score 107).

To analyze the influence of profession, gender and partner status, we performed variance analyses (ANOVA) (Table 4). While burnout, stress perception and perceived work burden scores were significantly different between women and men, gender associated differences were not found for CDI and its sub-constructs (only the *intensity* was marginally lower in men) (Table 4). Interestingly, MBI’s “Emotional Exhaustion” component did not significantly differ between women and men in general, but for profession. For profession, the strongest differences were found for MBI’s “Personal Accomplishment” component (Table 4). While the CDI did not significantly differ between nurses and physicians, instead burnout symptoms and perceived work burden were significantly higher in physicians, but not the stress perception itself (Table 4).

**Table 4:** Mean values of test variables within the sample

		CDI						MBI				PSS	WB
		frequency	intensity	sum score	PED	EWS		sum	EE	DP	PA		
All health care professionals	n	1335	1314	1310	1310	1310		1347	1347	1347	1347	1345	1347
	Mean	21.24	26.81	48.07	31.66	16.41		54.71	23.69	8.23	24.16	18.16	60.67
	SD	9.25	10.42	18.48	13.07	7.36		18.73	10.78	5.71	8.52	5.75	21.27
Gender													
Women	z-Mean ^a^	0.00	0.04	0.02	0.02	0.02		−0.07	−0.03	−0.13	−0.12	0.07	−0.08
	z-SD	1.00	1.01	1.01	1.02	1.00		1.00	1.02	0.98	0.96	1.00	1.01
Men	z-Mean ^a^	0.00	−0.09	−0.05	−0.04	−0.05		0.17	0.06	0.31	0.28	−0.14	0.19
	z-SD	0.99	0.96	0.99	0.96	1.01		0.97	0.94	0.97	1.04	0.97	0.94
F value		0.0	4.8	1.5	1.3	1.3		*16.8*	2.5	*58.5*	*47.3*	*12.2*	*21.0*
*P* value		n.s.	.028	n.s.	n.s.	n.s		*<.0001*	n.s.	*<.0001*	*<.0001*	*.001*	*<.0001*
Partner status													
Living without partner	z-Mean ^a^	0.14	0.13	0.13	0.12	0.12		0.02	0.05	0.06	−0.11	0.11	−0.02
	z-SD	1.04	1.01	1.02	1.02	1.04		1.02	1.02	1.04	0.94	1.06	0.98
Living with partner	z-Mean ^a^	−0.05	−0.04	−0.04	−0.04	−0.04		−0.01	−0.03	−0.02	0.04	−0.03	0.00
	z-SD	0.98	0.99	0.99	0.99	0.98		0.99	0.99	0.98	1.02	0.97	1.01
F value		*8.5*	*7.5*	*7.6*	6.6	6.7		0.3	1.7	1.9	5.5	5.3	0.1
*P* value		*.004*	*.006*	*.006*	.010	.010		n.s.	n.s.	n.s.	.020	.022	n.s.
Profession													
Nurses	z-Mean ^a^	−0.03	0.04	0.00	0.00	0.01		−0.29	−0.21	−0.28	−0.34	0.02	−0.10
	z-SD	1.01	1.02	1.01	1.02	1.01		0.92	0.97	0.92	0.87	1.01	1.03
Physicians	z-Mean ^a^	0.06	−0.07	−0.01	0.00	−0.03		0.57	0.41	0.56	0.69	−0.04	0.17
	z-SD	0.98	0.96	0.98	0.96	0.99		0.90	0.93	0.90	0.89	0.98	0.92
F value		2.3	3.4	0.1	0.0	0.5		*263.1*	*122.6*	*254.7*	*416.4*	1.1	*22.2*
*P* value		n.s.	n.s.	n.s.	n.s.	n.s.		*<.0001*	*<.0001*	*<.0001*	*<.0001*	n.s.	*<.0001*

*Abbreviations*: *CDI* Cool down Index, *CDI-PED* Perception of Emotional Distance, *CDI-EWS* Emotional Withdrawal as a Strategy, *MBI* Burnout, *EE* Emotional Exhaustion, *DP* Depersonalization, *PA* Personal Accomplishment, *PSS* Stress perception, *WB* Work burden

^a^standardized z-values; significant differences (*p*<0.01) were highlighted (italics)

With respect to the partner status (i.e., living with or without a partner), which might be seen as a resource, the CDI scored weakly higher in persons living alone, while in contrast the MBI scores did not differ between both partner status groups (Table 4). Instead, “Personal Accomplishment” was marginally lower in persons without a partner, and their stress perception was slightly higher, too.

### Predictors of cool down perceptions and reactions

In an attempt to identify which variables would predict health professionals’ Cool Down reactions best, we performed stepwise regression analysis for nurses and physicians separately (Table 5). All variables were included in the regression models which were either significantly related to the CDI or differed significantly in the aforementioned analyses, or due to theoretical considerations (i.e., influence of duration of work as a putative stressor).

**Table 5:** Predictors of Cool down reaction (stepwise regression analysis)

Dependent variable: CDI					Collinearity analyses	
	R^2^	Beta	T	p	Tolerance	VIP
Nurses						
F(6;746) = 169.2; *p* < .0001; Model 6: R^2^ = .57						
(constant)			6.182	<.0001		
Emotional exhaustion	51%	.401	10.325	<.0001	.377	2.654
Depersonalization	+3%	.207	7.415	<.0001	.725	1.378
Stress perception	+2%	.146	4.265	<.0001	.486	2.059
Work Burden	+0.8%	.107	3.592	<.0001	.644	1.554
Conscious Presence and Self control	+0.4%	−.062	−2.238	.026	.745	1.341
Team Satisfaction	+0.2%	−.056	−2.062	.040	.758	1.319
Physicians						
F(6;390) = 81.4; *p* < .0001; Model 6: R^2^ = .55						
(constant)			6.918	< .0001		
Depersonalization	44%	.411	8.943	< .0001	.540	1.852
Satisfaction with Team	+6%	−.154	−3.932	< .0001	.739	1.353
Emotional exhaustion	+3%	.160	3.223	.001	.461	2.171
Life Satisfaction	+1%	−.114	−2.628	.009	.604	1.656
Living with partner	+0.7%	−.096	−2.731	.007	.915	1.093
Work Burden	+0.7%	.101	2.546	.011	.730	1.369

The variables gender, self-efficacy expectation, work engagement, duration of work and stress perception were not significant in both models

For nurses, “Emotional Exhaustion” was CDI’s best predictor and explains 51% of variance. Three further variables would add 6% percent of explained variance, i.e., “Depersonalization”, stress perception and work burden. Two further included variables (i.e. situational awareness and team satisfaction) explain together <0.1% of variance and are thus not of strong relevance in this model.

For physicians, “Depersonalization” (44% of variance) was the best predictor. Team satisfaction would add further 6% of variance. Four further variables were significant in this model too, and would add additional 6% of explained variance, i.e. “Emotional exhaustion”, low general life satisfaction, living without a partner and work burden.

However, work engagement, self-efficacy expectation and gender were not among the significant variables in the respective models of nurses or physicians.

## Discussion

This study investigated Cool Down perceptions and reactions, burnout symptoms, and perceived work stress in a sample of nurses and hospital physicians. We assumed that all hospital staff experience Cool Down reactions to a similar degree, and the data confirm this with respect to profession and gender.

Our next hypothesis was that the perception of stress and work burden (incl. burnout symptoms) might be different between nurses and physicians and women and men. However, we assumed that these differences might not refer to Cool down reactions which are seen as an adaptive strategy. Also, this hypothesis can be confirmed, as in fact women and men differ significantly in the frequency of stress perception, work burden and burnout symptoms; with the exception of perceived stress the same significant differences were observed for physicians and nurses. In contrast, Cool Down reactions (which are the result of frequency and perceived intensity of respective perceptions and feelings) did not significantly differ with respect to gender and profession. This underlines that gender and profession related differences are of relevance for the *perception* of job demands and stressors, but not for the Cool Down reactions which are seen as an adaptive *strategy*. Our finding that MBI’s “Depersonalization” and “Personal Accomplishment” were significantly higher in male persons is (partially) in line with findings among nurses from Belgium that female nurses felt less “Emotional Exhaustion” and “Depersonalization” than male nurse [39]. However, Cool Down scores did not differ significantly for gender and profession. Even an exclusive focus on the *frequency* of Cool Down did not reveal significant differences between gender and professions, which are observable using the MBI.

The aforementioned gender and profession related differences seem to be attributed to the underlying concepts of CDI and MBI, which nevertheless share some similarities. Although CDI and MBI are strongly interrelated, exploratory factor analyses with both item pools show that the respective CDI and MBI subscales do not strongly intermix. This means, they can be regarded as more or less independent factors; they measure different aspects of the same ‘problem’.

We would conclude that nurses and hospital physicians are aware of their emotional distancing as a reaction towards stress and exhaustion, although particularly the physicians scored highest for (positive) “Personal Accomplishment” and (negatively rated) “Depersonalization” – but not for stress perception itself. This means, although the stress level may be similar, nurses and physicians perceived the *frequency* of their burnout symptoms differently, but they do not differ with respect to *frequency plus strength* of their Cool Down reactions as a strategy.

A further hypothesis was that work stress associated variables are positively associated with Cool Down reactions, while internal and external resources are negatively associated and might function as a buffer. Indeed, Cool Down was moderately to strongly related to measures of work stress (i.e., “Emotional Exhaustion” and “Depersonalization”, perceived stress and work burden) and negatively with staff’s resource variables (i.e., team satisfaction, work engagement, self-efficacy expectation and situational awareness), but not with duration of work per week which instead correlated with burnout symptoms. Also age and duration of employment were not significantly related with CDI scores, and only marginally with MBI scores.

Because we have found several variables with a significant influence on Cool Down reactions, it is important to investigate which of these would predict Cool Down best. Interestingly, the predictor pattern of Cool Down reactions is different for both professions. In nurses, “Emotional Exhaustion” was the best predictor (which explains 51% of variance), while in physicians “Depersonalization” (which 44% explained variance) was the best predictor of their CDI scores. For physicians further relevant predictors were “Emotional Exhaustion”, too, and satisfaction with the team and general life. In nurses, further relevant predictors were “Depersonalization”, too, and stress perceptions. This means, the inducing or aggravating influences may have a differential relevance for nurses and physicians, and this could be an effect of differences in the proportions of women and men in the samples. Indeed, it was not the “Emotional Exhaustion” which was perceived differently in women and men, but their *reaction* with respect to “Depersonalization” and “Personal Accomplishment” (which were higher in men and physicians).

With respect to our hypotheses that Cool Down reactions might be buffered by external and internal resources, we have to state that satisfaction with the team was found to be a negative (buffering) predictor only in hospital physicians (this resource was of marginal relevance in nurses and should thus not be overinterpreted). However, Conscious Presence and Self-Control (which can be seen as a measure of ‘natural’ situational awareness instead of ‘trained’ mindfulness) was of marginal relevance for nurses, too, while self-efficacy expectation (which was supposed to be a buffering factor) was not among the significant predictors for nurses and physicians. This does not mean that this variable is not of relevance – in fact it is moderately negatively related to Cool Down and burnout symptoms –, but of lower relevance in the respective models. Moreover, against our hypothesis, duration of work was not significantly related to Cool Down, but to burnout.

Surprisingly, lack of a supporting partner was associated with slightly higher CDI scores, but not with higher burnout scores or perceived work burden. One may assume that a partner is an external resource to give hold and emotional support, and that talking with the partner may help finding strategies to cope. However, this resource may buffer only the perceived *strength* of Cool Down, but not the *frequency* of the perceptions at all. In fact, the *frequency* of “Emotional Exhaustion” and “Depersonalization” did not differ between those with or without partner, but slightly their stress perception.

The above described pattern of predictors underlines that the individual reactions towards stress and the working environment are crucial for Cool Down perceptions and reactions. Longitudinal studies have shown that high social support may decease a person’s job dissatisfaction, while there is no interaction between demands, control and locus of control when social support is low at all [40]. In contrast, when social support is high, a person’s locus of control is of relevance for the balance of demands and control [40]. Thus, we have to assume a complex model of interacting variables which may change within time and demands. This has to be addressed in future longitudinal studies on the course of Cool Down reactions.

The idea that the emotional distance towards patients is an ‘effective’ strategy to protect health care professionals from work stress and burnout is actually a one-sided strategy; so far it may benefit only the staff and seems not to be as efficient as expected since it was proved that emotional distance may cause burnout [18]. Moreover, emotional distancing as a strategy will decrease also the quality of care, which in turns may result in higher dissatisfaction that one cannot hold the own ideals and standards of compassionate (and not only ‘functional’) care. Finally, perceiving that the own standards cannot be complied may result in higher frustration and burnout. In this sample, we can confirm that nurses’ satisfaction with the own ‘quality of care’ provided was moderately negatively related to CDI’s “Perceived Emotional Distance”, with burnout symptoms and with stress perception, and moderately positive with work engagement and best with nurses’ satisfaction with the team support.

Because all health care professionals may experience phases of emotional exhaustion and distancing, these reactions are not necessarily ‘pathological’. Yet, these reactions may result in different end-points, either times of sickness and finally inability to work (burnout), or finding of adaptive strategies to keep ‘functionality’ in the job (Cool Down). While burnout emerges gradually and evolves over time resulting in the onset of clinical relevant symptoms, compassion fatigue, a further related concept, occurs suddenly as a more acute beginning [19, 25, 41]. In contrast, Cool Down would be a subtle process of adaptation in response to the perceived exhaustion to retain functionality, and thus it may lack clinically relevant symptoms.

The underlying causes of this inner dissociation and strategic loss of empathic attention are diverse. In nurses, Spence Laschinga and Fida [42] found that both organizational and intrapersonal resources are important buffers against workplace stressors, burnout and mental health. They see the role of “authentic leadership” as important, but also strengthening positive intrapersonal resources [42]. Whether leadership and resilience programs that focus on individual rather than organization factors are in fact beneficial in the long-term has to be addressed in future studies. Or data support the hypothesis of van der Doef and Maes [14] that social support may buffer some of the negative influences of given stressors in so far as satisfaction with a supporting team in the hospital was moderately negative related to Cool Down reactions towards the stress perception.

### Limitations

A limitation of this study is its cross-sectional design which does not allow causal interpretations. So far we do not know whether the perception of an inner withdrawal in terms of Cool Down precedes the dysfunctional burnout trias, or whether they both may develop concordantly. Conceptually, their end-points are different. Future studies may rely on mixed methods and longitudinal design to further explore the differences between organizational and individual factors. So far it is also unclear whether persons suffering from dysfunctional burnout may finally chose distancing strategies when they return to their job; here, longitudinal studies are needed, too.

Moreover, we are aware that those with clinical relevant burnout symptoms may already have left their job or are at least not at work, and are thus not in the sample. We also have to state the possibility of omitted covariate bias; there might be further variables to explain the described variance in the regression models which remain to be identified in future studies.

A further limitation is that the sample of this study might not be representative for all nurses and hospital physicians, and should thus be regarded as a large convenience sample. Nevertheless, this sample seems appropriate to address the main research questions.

### Implications

The above described data would support the notion that health care professionals require a work environment which facilitates maintaining their motivation for an optimal and compassionate care of their patients. A structural approach of support by the hospital management would require first to control and eliminate work stressors (including work structure), and second a multifaceted approach to strengthen and support hospital staff’s resources in terms of team building, communication skills, resilience training, compassion training, etc. In nurses, both organizational and intrapersonal resources were identified as important buffers [42]. Resilience trainings for nurses to handle their stressful work environments were already tested [43], but remain to be implemented. So far it is unclear whether resilience and leadership programs that focus on individual rather than organization factors are indeed effective in the long term.

## Conclusions

Cool Down reactions were rather similar in women and men and nurses and physicians, but not their perception of burnout symptoms and perceived work burden which differed significantly. However, the predicting variables of Cool Down reactions were different, i.e. in nurses the best predictor was “Emotional Exhaustion”, while in physicians it was “Depersonalization”. It seems their perceptions are different, but not their adaptive Cool Down strategies to stay ‘functional’ in their job. These reactions may decrease the empathic (instead of an emotionally detached) care of the patient. Indeed, we found that in the sample of nurses their satisfaction with the own ‘quality of care’ provided was moderately negatively related with the perceived emotional distance and only marginally with an emotional withdrawal as a strategy. Thus, nurses are aware of these problems and are not satisfied with their reactions.

So far it is unclear whether the described distancing is in fact an active strategy or an unconscious reaction. Moreover, it remains to be shown whether Cool Down strategies are really effective to prevent burnout associated sickness and leaving the job. This has to be investigated in further studies.

## Additional file

Additional file 1: Questionnaire: Cool Down Index. (PDF 266 kb)

## Abbreviations

CDI: Cool Down Index
MBI: Maslach Burnout Inventor
