# Supplementary material for: Work stress associated cool down reactions among nurses and hospital physicians and their relation to burnout symptoms
Source: BMC Health Serv Res. 2017 Aug 10;17:551. doi: 10.1186/s12913-017-2445-3 (PMC5553651; doi:10.1186/s12913-017-2445-3)
Supplement: Additional file 1: — Questionnaire: Cool Down Index. (PDF 266 kb) [file 12913_2017_2445_MOESM1_ESM.pdf]

Cool Down Index
The following questions refer to your reactions and perceptions during your working day, especially with regard to the compassionate handling of persons you care for.<br>Please respond to these questions as honest as you can: There are no "right" or "wrong" answer.<br><br>Please rate the **frequency** of reactions and perceptions (“how often“) and also the **intensity** (“how strong”), and fill the respective number in the right columns..<br>• **How often**: 1 - a few times a year or less; 2 - once a month or less; 3 - a few times a month; 4 - once a week; 5 - a few times a week; 6 - every day<br>• **How strong**: 1 = very weak; (…) 4 = moderate; (…) 6 = very strong

In dealing with the people I look after (therapeutically), I notice that…	**How often?** (1-6)	**How strong?** (1-6)
… I simply must stop letting everything get to me to such an extent.		
… I have to withdraw with increasing frequency to protect myself.		
… their personal problems and worries often simply become too much for me.		
… I often no longer have the patience to listen to them.		
… I largely don´t care what they think of me.		
… I increasingly think how nice it would be to pack it all in.		
… some of them simply annoy me.		
… I myself increasingly go short.		
… I increasingly ‘work to rule’.		

CDI © Arndt Büssing, Witten/Herdecke University (2009); Contact: arndt.buessing@uni-wh.de
